# Comparison of muscle activity patterns of transfemoral amputees and control subjects during walking

**DOI:** 10.1186/1743-0003-10-87

**Published:** 2013-08-02

**Authors:** Eva C Wentink, Erik C Prinsen, Johan S Rietman, Peter H Veltink

**Affiliations:** 1 Faculty of Electrical Engineering, Mathematics and Computer Science, Biomedical Signals and Systems group, University of Twente, Drienerlolaan 5, Enschede 7500 AE, the Netherlands; 2 MIRA, Institute for Biomedical Technology and Technical Medicine, Drienerlolaan 5, Enschede 7500 AE, the Netherlands; 3, Roessingh Research and DevelopmentRoessinghsbleekweg 33b, Enschede 7522 AH, the Netherlands; 4 Biomechanical Engineering group, University of Twente, Drienerlolaan 5, Enschede 7500 AE, the Netherlands

**Keywords:** EMG, Transfemoral amputee, Kinematics, Spatio-temporal data

## Abstract

**Background:**

Only few studies have looked at electromyography (EMG) during prosthetic gait. Differences in EMG between normal and prosthetic gait for stance and swing phase were never separately analyzed. These differences can give valuable information if and how muscle activity changes in prosthetic gait.

**Methods:**

In this study EMG activity during gait of the upper leg muscles of six transfemoral amputees, measured inside their own socket, was compared to that of five controls. On and off timings for stance and swing phase were determined together with the level of co-activity and inter-subject variability.

**Results and conclusions:**

Gait phase changes in amputees mainly consisted of an increased double support phase preceding the prosthetic stance phase. For the subsequent (pre) swing phase the main differences were found in muscle activity patterns of the prosthetic limb, more muscles were active during this phase and/or with prolonged duration. The overall inter-subject variability was larger in amputees compared to controls.

## Background

During rehabilitation transfemoral amputees learn to adapt their gait pattern to walk with a prosthesis. Several of these adaptations are already known. During gait the stance phase of the amputated limb shortens compared to that of the intact limb. Therefore the swing phase is longer for the amputated limb. The double support phase elongates when the amputated limb becomes the stance limb and shortens when the intact limb becomes the stance limb [1,2]. The comfortable walking speed of prosthetic walkers is also lower than in normal walking [1,3-5]. Kinematic data shows that transfemoral amputees lack plantar flexion power (push-off) at the prosthetic side. To facilitate forward propulsion they increase the work by the hip joint at the prosthetic and intact side and the plantar flexors at the intact side [4-6]. Eccentric work at the hip of the intact side decreases with respect to normal gait [5]. Joint power during concentric knee extension increases for the intact side, with respect to normal walking [4].

However, little research has been performed on electromyography (EMG) during amputee gait. EMG of residual limb muscles of TFA may give valuable information on adaptations besides those that can already be found using the kinetic and spatio-temporal data [7]. Some studies report increased and prolonged muscle activity in amputees during gait [3,6,8]. Bae et al. [8] concluded that the co-activation of the upper leg muscles of the intact limb in amputees was larger than in controls. Hong and Mun [9] found that during gait the muscle activity of residual limb muscles in TFA is correlated to the socket pressure. If EMG patterns are different from that of controls this might indicate specific adaptations of amputees. Muscle activity per phase (stance and swing) can give more insight in the changes in the muscle activity patterns, how they change compared to normal walking and in the adaptations amputees make when walking with a prosthesis, besides kinematic changes.

In the current study we focus on muscle activity during the stance and swing phase of prosthetic gait. Do the muscle activity patterns of the prosthetic limb change and how do they change for the stance and swing phase, compared to normal gait? We intended to have as little interventions to the prosthesis and the subsequent walking pattern as possible. Therefore we measured EMG inside the socket, without modifications, of six amputees and compared this to data of five controls. Previous studies have shown that it is possible to measure EMG with acceptable quality inside the socket of amputees [3,10].

From this data we determined if the timings of the muscle activity changed with respect to the different phases of gait compared to normal gait. We hypothesized that the general EMG patterns during walking are comparable to those in controls, but we expected to find differences related to specific adaptations in amputees. Three muscles at the contralateral lower leg were also measured to determine the adaptations at the intact lower leg. We determined how the inter-subject variability of amputees compares to that of controls. Spatio-temporal and kinematic data were also measured for gait phase determination and to relate the results to other studies to determine the gait phases and to compare the results to previous studies.

## Methods

### Participants

Eleven healthy subjects participated in the study, five controls and six unilateral amputees. All subjects were recruited between April and July 2011. Of the amputees there were three transfemoral amputees (TFA) and three through the knee amputees (TKA). An overview of the amputees can be found in Table 1. Inclusion criteria were: have a unilateral TFA or TKA regardless of the reason for amputation; be between 18 and 70 years old; be a prosthetic user able to walk independently with or without a walking aid (K-level 2, 3 and 4). The controls were on average aged 23 (range 21-27) and had no history of lower leg injuries, neurodegenerative diseases or any skin conditions. An informed consent was obtained before the experiments, and the study was approved by the local Ethics Committee. The institutional board, for the approval of the study is called METC Twente (or Medisch Ethische Toetsingscommissie Twente).

**Table 1:** Overview of the details of the amputees

**Subject**	**Type**	**Age**	**Reason**	**Residual limb**	**Knee**	**Foot**	**Time**
		**(years)**	**amputation**	**length(m)**			**(months)**
1	TKA	52	T	0.56	C-leg	C-walk	24
2	TKA	46	T	0.59	Rheo knee	Vari-Flex Evo	8
3	TKA	29	D	0.56	C-leg	1E56	5
4	TFA	61	Vas	0.41	Total knee	Elation	5
5*	TFA	64	Vas	0.41	Total knee	Elation	6
6	TFA	62	T	0.35	C-leg	1E56	133

(T = trauma, D = post-traumatic dystrofy, * walked with walking aid, time since amputation).

### Measurements

EMG recording was performed on eight upper leg muscles in all subjects: m. gluteus maximus (GMa), m. gluteus medius (GMe), m. tensor fasciae latae (TFL), m. rectus femorus (RF), m. vastus lateralis (VL), m. biceps femoris (BF), m. semitendinosis (ST), m. adductor magnus (Add). In amputees these were measured on the residual limb, in controls these muscles were measured at one limb, which was alternated between dominant and non-dominant limb. For amputees and controls this limb will be called the “prosthetic limb” and “mimicked prosthetic limb” respectively.

At the contralateral lower limb three more muscles were measured, the m. tibialis anterior (TA), m. gastrocnemius medialis (GaM) and the m. soleus (Sol). For amputees and controls this limb will be called the “intact limb” and the “mimicked intact limb” respectively.

Electrodes were placed according to the SENIAM standards [11], by an experienced physical therapist. For the amputees the locations were approximated, but EMG was checked prior to the measurements by selective contraction of the muscle [11]. On each muscle two self adhesive electrodes (Ambu, BRS) were placed as closely together as possible. EMG measurements were performed with a 16 bipolar channel Porti-system (TMSi, Oldenzaal, the Netherlands) at a sample frequency of 2048Hz, no pre-filtering was applied.

Footswitches, placed mid-heel and under the first metatarsal head of each the foot, gave information about initial contact and initial swing. Footswitch data was registered with the Porti-system.

Kinematic data were measured (100Hz) using inertial sensors from Xsens (Xsens, Enschede, the Netherlands), with 3D accelerometers, 3D gyroscopes and 3D magnetometers. Two inertial sensors were placed at the upper and lower (mimicked) prosthetic limb. Subjects wore their own low-heeled shoes.

To synchronize EMG, footswitches and inertial sensors a synchronization pulse (sync) was given at the start and end of each measurement which was visible in all data sets.

### Procedures

For the experiments the subjects were asked to walk at a self selected walking speed. After data recording was started, the sync was pressed and subjects started walking. After five steps they were asked to stop, turn around, wait 2-3 seconds, press the sync and walk back; this constituted one trial. Four trials were performed in all subjects.

### Data analysis

From the footswitch data the timings of initial contact (IC), terminal stance, initial swing and loading response of each limb were determined [12]. Foot switches were used to extract the spatio-temporal information. Full strides were cut from the EMG and inertial sensor data, from IC to IC of the (mimicked) prosthetic limb. Strides with gait initiation or termination were excluded. All strides per subject were aligned at IC of the (mimicked) prosthetic limb.

#### ***Inertial sensor data***

The inertial sensor data was expressed in the body coordinate system based on a sensor-segment calibration procedure as described by Wentink et al. [13]. This data was subsequently low-pass filtered at 10 Hz with a second order, butterworth filter. From the calibrated inertial sensor data the knee angle, hip adduction and abduction are calculated using accelerometer and gyroscope data by the method described by Watanabe et al. [14].

#### ***EMG data***

**Figure 1:**
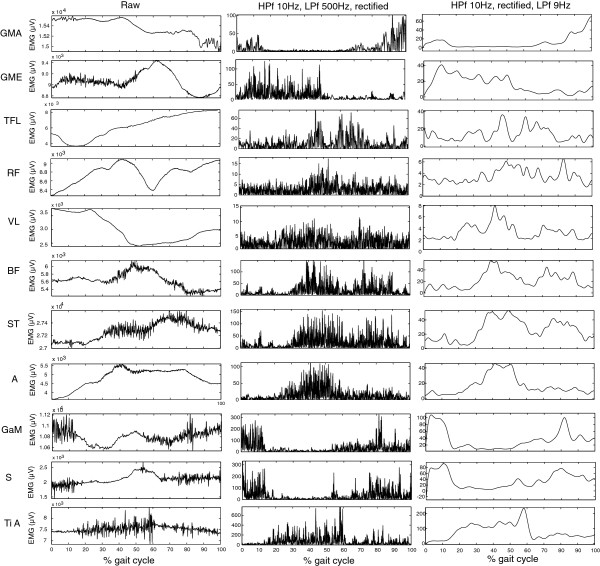
**Raw EMG data.** An example of filtered activity of all upper and lower leg muscles, of one subject during one trial. The row on the left shows the raw data of the trial during one gait cycle. The middle row shows the high pass filtered (HPf), rectified and low-pass filtered (LPf) data of the same trial and the right hand row shows the linear envelope HPf at 10Hz, rectified and LPf at 9Hz.

**Figure 2:**
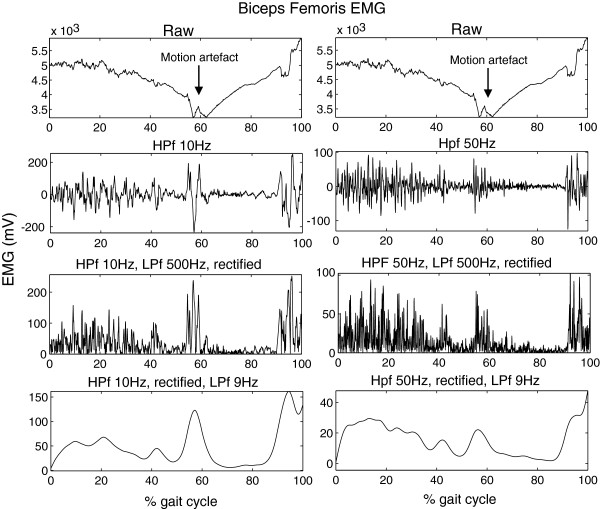
**Motion artifacts.** An example of one amputee of the Biceps Femoris EMG with a motion artifact. On the left the data is high-pass filtered at 10Hz, as in all trials without motion artifacts, but this does not remove the artifacts. On the right the data is high-pass filtered at 50Hz, which did remove the motion artifact. Trials with this type of artifact that was removed by a 50Hz HP filter, but not by a 10Hz Hp filter were removed from the data. Most trials did not show this type of artifact and therefore the trials with artifacts were removed from the analysis and the original filtering was used.

**Figure 3:**
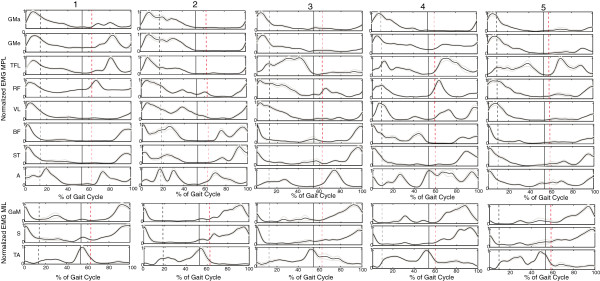
**Ensemble averages controls.** The ensemble averages of each of the controls averaged over all trails (20) for each of the measured muscles. The black dashed line represents initial swing of the MIL, the black solid line initial contact of the MIL and the red dashed line initial swing of the MPL. (MPL = mimicked prosthetic limb, MIL = mimicked intact limb).

**Figure 4:**
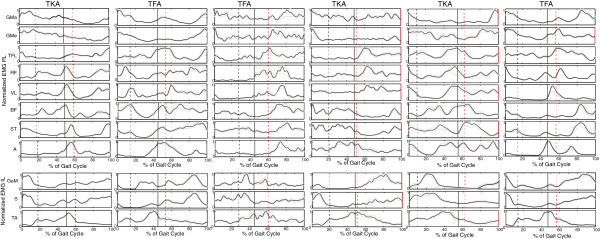
**Ensemble averages amputees.** The ensemble averages of each of the amputees averaged over all trails (20) for each of the measured muscles. The amputee data is deliberately not placed in the order of amputees seen in Table 1, to prevent matching of data and subjects. The black dashed line represents initial swing of the IL, the black solid line initial contact of the IL and the red dashed line initial swing of the PL. (PL = prosthetic limb, IL = intact limb).

EMG data was first high pass filtered at 10Hz and subsequently low pass filtered at 500Hz, both with a second order butterworth filter. In Figures 1, 2, 3, and 4 the ensemble averages of all amputees and controls separately are provided, including the raw and filtered data of one subject, of one trial for all muscles. In Figure 1 an example of filtered data is presented. For on and off detection the data was rectified and integrated (IA) in a window of 20 samples, a post processor of 4 windows was used. The threshold for on/off detection was determined per muscle. A period of rest activity was selected, and the mean IA value of this period plus three times the standard deviation was used as threshold for onset and termination of muscle activity [15-17]. For each muscle, each stride and each subject the on/off timings were calculated. These timings were averaged per subject, to get the on and off timings per muscle, per subject.

The stance and swing phase of the (mimicked) prosthetic limb were calculated per subject and expressed as percentage of the total stride time. Using the average muscle on/off timings per subject, we subsequently calculated for which percentage of the stance or swing phase the muscles were active. These were subsequently averaged for the controls and the amputees. Differences between controls and amputees were analyzed using a Kruskal-Wallis test. The level of alpha was set at 0.05.

The inter-subject variability of the EMG data was determined using the variance ratio (VR) for each subject and muscle for the stance and the swing phase [17,18]. The VR is the variance of the data between gait cycles normalized to the total variance, whereby 0 indicates a low variance and 1 a high variance. Differences between the controls and amputees were analyzed using the Mann-Whitney-Wilcoxon test [17]. The standard error of the mean (SEM), was calculated using $\text{SEM}=\frac{{\text{SD}}_{\mathrm{\%on}/\text{offtime}}}{\sqrt{N}}$, where N is the number of subjects per group [19].

## Results

### Kinematic data

**Figure 5:**
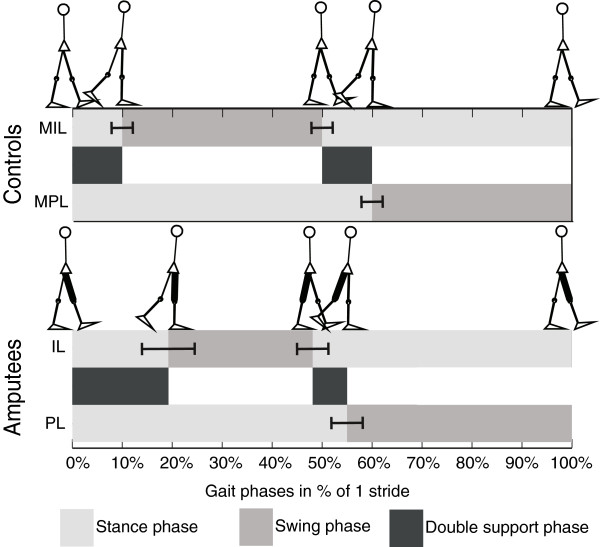
**Gait phases.** Gait phases for controls and amputees, as percentages of one full stride. In black the swing phase, in light grey the stance phase and in dark grey the double support phases. The whiskers give one SD.

In Table 2 the average duration of a stride and the different gait phases in percentages of a stride are presented. A shift of all phases can be seen for amputees, Figure 5. For amputees the relative duration of the stance phase of intact limb, the prosthetic swing phase and the (first) double support phase before the prosthetic single stance phase are significantly increased compared to controls. The (second) double support phase of amputees before the prosthetic swing phase, is shortened but not statistically significant. Compared to the total stance phase, this “second” double stance phase is equal for both controls and amputees (15%). No differences were found between TFA and TKA, nor between mechanical and micro-processor-controlled (MPC) knees.

**Table 2:** Gait phases

**Phase**	**Controls**	**Amputees**	**Statistical note**
	**value (SD)**	**value (SD)**	
Stride duration	1256ms (72)	1468ms (307)	NS
Total stance (M)PL	61% (2)	55% (9)	C >A p =.010
Total stance (M)IL	60%(3)	71% (6)	C <A p =.008
Swing (M)PL	39% (2)	45% (3)	C <A p =.010
Swing (M)IL	40% (3)	29% (3)	C >A p =.008
DLS (M)PL	11% (1)	20% (9)	C <A p =.045
DLS (M)IL	10% (3)	7% (3)	NS

(C = controls, A = amputees, NS = not significant, (M)IL = (mimicked) intact limb, (M)PL = (mimicked) prosthetic limb, SLS = single limb support, DLS = double limb support).

#### ***Joint rotations***

Table 3 shows the movements in degrees around the hip and knee the movement patterns around the hip and knee joints. Hip adduction and abduction are significantly reduced in amputees compared to controls. Knee flexion during stance as well as swing is also significantly reduced in amputees. No differences were found between TFA and TKA, nor were they found between mechanical and MPC knees.

**Table 3:** Rotations of knee and hip

	**Controls**			**Amputees**		**Statistical note**
	**Average (SD)**	**Range**		**Average (SD)**	**Range**	
Max Hip flexion	28°(5)	19°- 37°		26°(12)	15°- 43°	NS
Max Hip extension	13°(5)	6°- 20°		15°(8)	2°- 25°	NS
Max hip adduction	9°(1)	8°- 10°		6°(2)	3°- 9°	C >A p =.006
Max hip abduction	11°(2)	9°- 14°		7°(3)	3°- 11°	C >A p =.002
Max knee flexion stance	13°(4)	8°- 18°		4°(3)	0°- 6°	C >A p =.006
Max knee flexion swing	57°(6)	47°- 68°		42°(13)	22°- 55°	C >A p =.029

(C = controls, A = amputees, NS = not significant).

### EMG data

**Figure 6:**
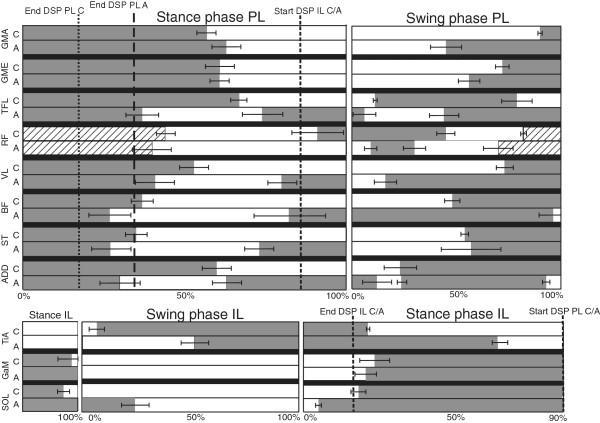
**Overview muscle activity.** The average muscle activity of all muscles, for controls and amputees, as a percentage of the (mimicked) prosthetic stance and swing phase. In dark grey the muscle is “on”, in white it is “off” and in hatched white periods of possible cross-talk. For the stance phase 0% is IC of the (mimicked) prosthetic limb and 100% is initial swing of the (mimicked) prosthetic limb. The end of the first double stance phase (DLS PL) before (mimicked) prosthetic stance (PL), of controls and amputees are indicated. The start of the second double limb support (DLS IL) is also indicated, which is equal for controls and amputees. For the swing phase, 0% is initial swing of the (mimicked) prosthetic limb and 100% is IC of the (mimicked) prosthetic limb. The whiskers show the standard error of the mean (SEM). All upper leg muscles are measured at the (mimicked) prosthetic limb and all lower leg muscles at the contralateral (mimicked) intact limb. Lower leg activity is scaled similarly, but to the phases of the intact leg. Hereby part of the stance phase (the DLS PL) is placed at the left hand side of the figure.

In Figure 1 a sample trial of EMG measured inside (upper leg) and outside (lower leg) the socket are provided of one subject. Both EMG measured inside and outside the socket shows to be of similar quality, without motion artifacts. 24 complete steps were measured in each subject, per subject at least 20 steps were included in the analysis. No steps were excluded from the controls. From the amputee data four subjects showed motion artifacts (see example in Figure 2) in maximally three trials in one or more muscles. In one other amputee four trials were excluded due to missing footswitch data. Figures 3 and 4 show the ensemble averages of each of the controls and amputees respectively. Figure 6 shows the timings of the upper leg muscles of the (mimicked) prosthetic limb for amputees and controls as percentages of the stance and swing phases of the (mimicked) prosthetic limb. Data of the lower leg muscles are from the contralateral limb.

#### ***Stance phase***

During the (prosthetic) stance phase, the GMa of the amputee group is active for a longer period after initial contact. All other upper leg muscles are active for a similar or shorter period. In the amputee group, some muscles, become active a second time during stance; the TFL, VL, BF, ST and Add. In the controls this second phase of activity during stance for these muscles is not seen. The first period of activity shown for the RF is probably crosstalk by the VL [20,21]. The RF becomes active just before terminal stance in controls, but shows no activity in this phase in the amputee group.

During the stance phase of the intact limb, activity of the GaM starts around the same time in controls and amputees, Sol activity of amputees starts a little earlier. The activity of the TiA in amputees continues longer during the stance phase of the intact limb, compared to controls. No significant differences were found between the activation patterns of the stance phase between controls and amputees.

#### ***Swing phase***

The differences in muscle activity during the swing phase are larger than for the stance phase. The GMe and GMa of the amputees become active in the second half of the swing phase, whereas in the controls they become active at the end of the swing phase. This increased duration of activation is also seen for the RF, the VL and the BF. The TFL is also active at the transition from stance to swing, and has a later “second” activity onset at the end of the swing phase. The Add is active in amputees before initial swing and at the beginning of the swing phase, which is not the case in controls.

TiA activity during the swing phase of the intact limb starts later in amputees compared to controls. No differences are seen in GaM activity during the swing phase of the intact limb, but the Sol of the amputees shows activity during the first part of the swing phase, where controls do not show this activity. No significant differences were found between the activation patterns of the swing phase of controls and amputees.

#### ***Variability***

**Figure 7:**
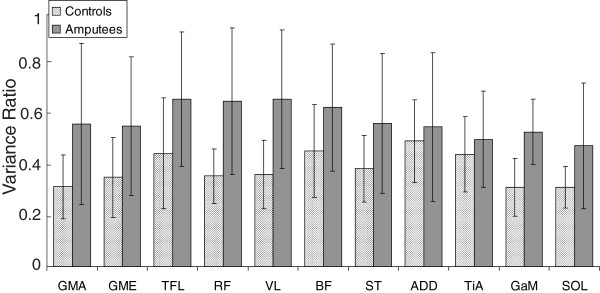
**Variance ratios.** The VR of all muscles of controls and amputees. Whiskers give one SD.

The overall inter-subject variability of the EMG data of amputees is significantly lower (p = 0.011) than that of controls (Figure 7). The variability per muscle however is in none of the muscles significantly different. The mean VR of controls ranged from 0.30 to 0.48, for amputees this range was 0.47 to 0.64. The SEM of the EMG data is around 3% of the stance and swing phase for controls, for amputees this is a little higher, around 4% of the stance and swing phase with some outliers at 12-14%.

## Discussion

### Kinematic and spatio-temporal data

The kinematic data showed that the stance phase duration of the intact limb increases and the prosthetic swing phase duration also increases in amputees. This coincides with the general concept that amputees tend to stand longer on their intact limb than on their prosthetic limb, which has also been found in other studies [1,2]. Knee flexion during initial stance differs. Controls show a knee flexion of up to 18°, in amputees this is only 4° even though all amputees had a prosthetic knee which allows knee flexion during stance. This lack of knee flexion might indicate that amputees are not comfortable using knee flexion during initial stance of the prosthetic limb, which may be caused by a lack of trust or experience in using the MPC knee to the full potential. Hip adduction and abduction are also reduced in amputees, which was also reported by Jaegers et al. [1]. The reduction in hip adduction has most likely only a small effect on the walking pattern, as it is only a few degrees less than in controls. During normal single limb stance a small amount of adduction is seen, to ensure that the center of mass does not have to move laterally to keep it above the supporting surface. However when amputees are in prosthetic single limb support they will not bring their COM above their support surface, but keep it more medially. This can be explained by the fact that in the frontal plane they have little opportunity to correct themselves, too much lateral motion will cause a fall. This reduces the need for adduction in stance. The reduced abduction may change the walking pattern of amputees. Hip abduction is used to “shorten” the leg to ease foot clearance during the transition from the stance to the swing phase. However TFA generally find it more difficult to perform hip abduction, which makes foot clearance more difficult. The reduction in hip abduction may create the need for more adaptations from the intact limb, for instance increased plantar flexion during single intact limb support (vaulting).

### EMG

The differences found in muscle activity between prothetic users and controls are mainly present in the (pre)swing phase. Muscle activity of controls resembles that of previous studies, although muscles show activity for a longer period of time [12,22]. This may be due to the onset detection method, but the exact methods used in the previous studies were not described. Therefore it is hard to find a clear explanation for this discrepancy. Visual comparison of raw and filtered EMG data showed comparable EMG quality between controls and amputees.

#### ***Stance phase***

When the gait stance and swing phases are compared separately, muscles in amputees do not seem to be active for much longer than in controls. At the end of the stance phase a period of activity is seen in most of the upper leg muscles, starting around the beginning of the second double support phase. This may be the mechanism by amputees to increase socket fitting at the end of the stance phase, to prepare for lifting of the prosthesis in the swing phase [9]. Lower leg muscles of the contralateral side show a prolonged activity during stance. This increased activity could be used to ease foot clearance, ankle plantar flexion of the intact limb is used to virtually lengthen the intact limb. The prolonged activity can also be explained by the increased push-off needed from the intact limb, to propel the body forwards, to compensate for the lack of push-off on the prosthetic side. This coincides with the kinetic data, which showed increased work at the hip and plantar flexors of the intact limb [4-6].

#### ***Swing phase***

Some of the upper leg muscles of the amputees, the BF and the VL, remain active for almost the complete swing phase. The other muscles all become active again at the end of the swing phase to prepare for initial contact. These muscles show an earlier activity onset than in controls, which may be explained by the walking strategy of amputees. Many amputees try to fully extend the knee to ensure it is locked at the end of the swing phase which is also confirmed by the reduced knee flexion during initial stance.

Our results resemble the results presented by Jaegers et al. [3], as far as they can be compared. They only showed muscle activity for the complete gait cycle and no exact onset timings were calculated. They also reported activity before initial swing and found differences between subjects with an amputation in the proximal or distal half of the upper leg [3]. In the current study all amputees were amputated at the distal half of the upper leg. In some muscles the activity is slightly longer or shorter compared to Jaegers et al. [3]. This can be due to the separated stance and swing phases in the current study and due to different approaches in detection times.

TFA showed a different activation pattern in some phases of the gait cycle, which shows that they adapt to their new prosthetic situation. Although the results show that consistent muscle activity can be measured inside the socket of TFA, the usability for prosthetic control is questionable. Variability between the amputees is higher, although patterns within the amputees are consistent. Although muscle activity patterns can change due to the disturbed anatomy by the amputation and by use of the prothesis, training may allow TFA to learn new walking patterns which in turn may need adaptations in the muscle activity patterns to control a prosthesis [10].

#### ***Variability***

The overall inter-subject variability of the EMG data from the amputees was significantly higher than that from controls. VRs per muscle were however for none of the muscles significantly different. Granata et al. [23] reported VRs in healthy adults between 0.17 and 0.27, although they can go up to 0.76 in healthy adults [24]. The main reason for a higher VR in amputees (up to 0.64) is most likely the lower walking speed [25].

Many of the muscles in the upper leg of the amputees are cleaved. The electrodes were placed and tested for activity according to the SENIAM standards. However, due to the amputation some muscles may have a different location and the location of the electrodes may not have been ideal. Rotations in the socket may also affect the position of the electrodes with respect to the muscle. Poor socket fitting will affect the repeatability of the signal, this will induce more noise and the prosthetic user may show more muscle activity to properly control or fit the prosthesis. One subject complained of non-optimal socket fitting as it was too large. This subject had a higher VR. None of the subjects complained about the EMG electrodes, they did not seem to effect the socket fitting. However, this does not explain the increased VR in the lower leg muscles of the contralateral limb and the hip muscles. This might indicate that the walking pattern of amputees is less consistent than that of controls. The standard deviations within amputees for spatio-temporal and kinematic data were also larger than in controls.

### Methodological considerations

The amputees were a mixed group. No inclusion criteria for type of amputation or time since amputation were added. The average age (52.3) was larger than that of the controls (23). Previous studies have shown that aging may affect the spinal cord activity, walking speed and cause a higher spread in muscle activation [22,26]. Also three prosthetic users were only prosthetic users for 5-6 month, of whom the EMG pattern may still change over time. Two of them were the elderly subjects with vascular diseases. One of these subjects also walked with a walking aid, which may also effect the muscle activity [27], both subjects had higher VRs. Nevertheless, even with the large variability in the group, no large deviations were seen in the EMG patterns of these subjects.

A more homogeneous and larger group of amputees with similar prosthesis may reduce the variability between the subjects. We did not find any obvious differences between the different knees, but this may also be caused by the low number of amputees. Including amputees with a short residual limb as Jaegers et al. [1,3] did, can be an interesting addition. Measurements were performed inside the socket of the amputees. The residual limb-socket interface may have lead to increased motion artifacts, compared to using an experimental socket with build in EMG sensors. Data were checked for these artifacts. It occurred only occasionally during initial contact or initial swing that these artifacts were not removed by filtering. Trials with motion artifacts were removed, but this still allowed at least 20 steps to be included per subject. No motion artifacts were found in controls. Although we measured EMG inside the socket with reasonable quality, we did not test the reliability and validity compared to EMG measured using an experimental socket. We placed electrodes and performed EMG measurements according to the SENIAM standards, which are based on normal anatomy. No information on actual muscle locations were available, for instance from MRI. After electrode placement muscle activity was checked using selective muscle contraction. Only occasionally electrodes needed to be replaced, for a better location with respect to the muscle belly, but never more than 2-4 cm from the original placement. Therefore normal anatomy was assumed in amputees, with respect to cross-talk. Surface EMG was used for ease of electrode placement and comfort to the patient. Intramuscular EMG may have given less cross-talk and possibly more information on specific muscle activity, but it is impossible to use in the own socket of the amputees and very uncomfortable to the patient.

## Conclusion

In amputees the double support phase before the prosthetic stance phase increases significantly and the prosthetic swing phase shortens. EMG patterns mainly differ at the end of the stance phase and in the swing phase. These changes can explain the changes in walking strategy, but are likely also required to improve socket fitting. In this study EMG was measured inside the socket of amputees, and the data showed to be of comparable quality compared to that of controls. Variance within each amputee is higher than in controls, but variability in the kinematic data between the amputees is also higher. The increased variance may mainly be caused by the variability in walking pattern and cleavage of muscles.

## Competing interests

The authors declare that they have no competing interests.

## Authors’ contributions

EW was involved in the study design, the setup of the measurement protocol, the measurements, the data analysis, writing and revision of the manuscript. EP participated in the setup of the measurement protocol, the measurements, drafting, and revision of the manuscript. HR contributed to the study design and drafting of the manuscript. PV participated in the study design, the data analysis and drafting of the manuscript. All authors read and approved the final manuscript.
